# Healthy lifestyle and Alzheimer’s disease in individuals with hyperlipidemia: A prospective cohort study

**DOI:** 10.1016/j.tjpad.2026.100520

**Published:** 2026-02-27

**Authors:** Danyang Sun, Linling Yu, Chenqi Liao, Yuzhong Xu, Wei Liu, Xiong Wang

**Affiliations:** a Department of Laboratory Medicine, Tongji Hospital, Tongji Medical College, Huazhong University of Science and Technology, Wuhan 430030, China; b Department of Public Health, Tongji Hospital, Tongji Medical College, Huazhong University of Science and Technology, Wuhan 430030, China; c The Baoan People’s Hospital of Shenzhen, The Second Affiliated Hospital of Shenzhen University, Shenzhen 518101, China

**Keywords:** Alzheimer’s disease, Hyperlipidemia, Healthy lifestyle, Disease prevention

## Abstract

**Background:**

Whether healthy lifestyle behaviors are associated with Alzheimer’s disease (AD) risk among individuals with hyperlipidemia remains unclear.

**Methods:**

We analyzed 241,642 dementia-free participants from the UK Biobank. A weighted lifestyle score (0–7) was derived from seven factors and categorized into five tiers. Hyperlipidemia was defined as lipid-lowering medication use or LDL-cholesterol ≥ 4.0 mmol/L. Cox regression estimated hazard ratios (HRs) and 95% confidence intervals (CIs).

**Results:**

Over a median follow-up of 14.5 years, 1728 AD cases occurred, including 977 cases among 104,082 individuals with hyperlipidemia. Compared with the intermediate tier, unhealthy lifestyle was associated with elevated AD risk (HR: 1.17; 95% CI: 1.02–1.35), while healtshy and very healthy tiers were associated with progressively lower risk (HR=0.85 and 0.74, respectively). These associations were evident among individuals with hyperlipidemia, but not statistically significant among those without hyperlipidemia.

**Conclusions:**

Healthy lifestyle patterns were associated with lower AD risk among individuals with hyperlipidemia, with greater risk reductions observed for healthier lifestyle tiers.

## Highlights

• Healthier lifestyle patterns were associated with progressively lower AD risk in the overall cohort.
• This protective association was observed predominantly among individuals with hyperlipidemia.
• The benefits were most pronounced in hyperlipidemic individuals aged >60 years.
• These associations remained consistent across genetic risk strata and AD polygenic risk score.

## 1 Introduction

Alzheimer’s disease (AD) represents the leading cause of neurodegenerative dementia globally, manifesting as gradual cognitive deterioration that ultimately compromises independent functioning [1]. Its neuropathological features include extracellular amyloid-β (Aβ) plaques and intraneuronal neurofibrillary tangles composed of hyperphosphorylated tau protein [2]. In the absence of curative treatment, identifying modifiable risk factors and preventive strategies remains a public health priority.

Hyperlipidemia involves abnormalities in circulating lipid profiles, most notably reflected in shifts in the levels of atherogenic lipoproteins and cardioprotective lipoprotein fractions [3]. Growing research further indicates that lipid abnormalities extend beyond cardiovascular disease and may also contribute to the development of AD [4]. Cholesterol, a critical membrane component essential for neuronal plasticity, may influence AD pathogenesis by modulating the lipid microenvironment in which β- and γ-secretases cleave amyloid precursor protein (APP) to generate Aβ [5].

Apolipoprotein E (APOE) serves as the principal apolipoprotein within the brain, residing predominantly in high-density lipoprotein particles and mediating cholesterol shuttling from glial cells to neurons [6]. Genome-wide association study (GWAS) has identified APOE as the first gene associated with late-onset AD, establishing it as a key lipid-related factor in AD pathogenesis [7]. Moreover, multiple genes involved in lipid metabolism have likewise been linked to AD, including SORL1 [8], CLU [9], and ABCA7 [10]. Collectively, these findings support a role of lipid dysregulation in AD.

Given that hyperlipidemia is common and modifiable, preventive strategies such as lifestyle interventions may offer substantial benefits for reducing AD risk. Healthy behaviors, including not smoking, moderating alcohol consumption, engaging in physical activity, adhering to a cardioprotective diet, obtaining adequate sleep, minimizing sedentary time, and maintaining social engagement, are linked to reduced cardiovascular and dementia risk [11]. Smoking and physical inactivity increase the risk of AD, while favorable sleep duration and regular social contact have been associated with better cognitive outcomes [12,13].

Hyperlipidemia and AD share common lifestyle-related risk factors, suggesting that lifestyle modification may benefit both cardiovascular and cognitive health. However, most studies have examined single lifestyle behaviors in isolation, with few evaluating composite lifestyle scores in relation to dementia risk [14,15]. Importantly, individuals with hyperlipidemia represent a high-risk yet potentially modifiable population, but it remains unclear whether adherence to multiple healthy lifestyle behaviors can mitigate AD risk within this subgroup.

Leveraging UK Biobank data, we constructed a multidimensional lifestyle score incorporating seven factors and examined its association with incident AD, with particular focus on whether these associations differ by hyperlipidemia status, age, and genetic susceptibility. We therefore aimed to construct a multidimensional lifestyle score and examine its association with incident AD using UK Biobank data, with particular attention to potential effect modification by hyperlipidemia status and genetic susceptibility.

## 2 Methods

### 2.1 Study population

The UK Biobank comprises a population-based prospective investigation enrolling approximately 500,000 adults aged 37–73 years during 2006–2010. Enrollees completed baseline questionnaires, underwent clinical evaluations, and donated biospecimens[16]. Written consent was obtained universally, with ethical oversight provided by the North West Multi-Centre Research Ethics Committee[17]. The present work was conducted under application ID 106528.

**Fig. 1:**
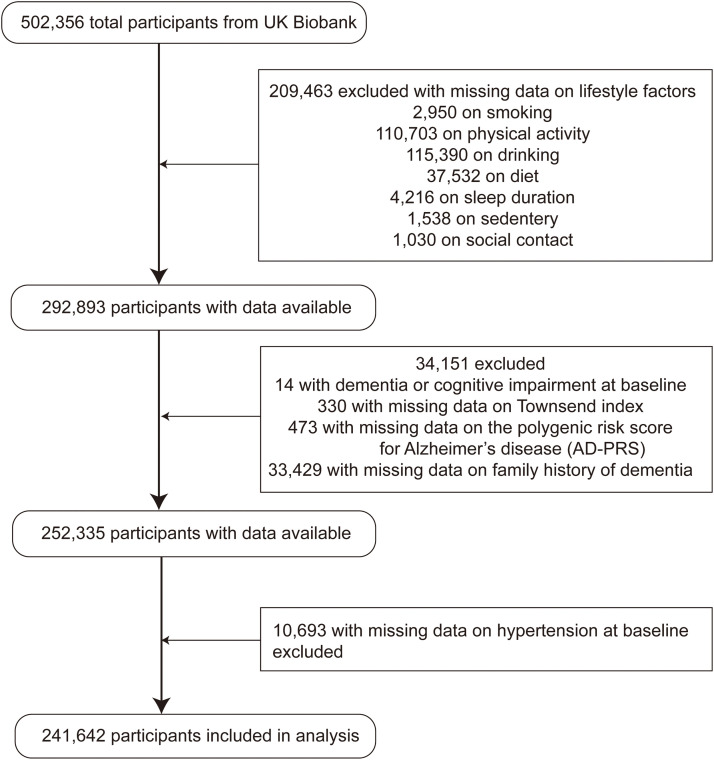
Flow chart for the study sample from the UK Biobank.

Participants with dementia at baseline (including senile cerebral degeneration or other neurodegenerative diseases) were excluded, as were those with missing data on the seven lifestyle factors, AD polygenic risk score (AD-PRS), or Townsend deprivation index (TDI). Missing values for other covariates were handled using multiple imputation. The final analysis included 241,642 baseline dementia-free individuals. The participant selection process is illustrated in Fig. 1.

### 2.2 Assessment of healthy lifestyle

Healthy lifestyle was assessed using seven lifestyle factors selected based on prior evidence linking them to dementia risk: four traditional cardiovascular factors (smoking, alcohol intake, physical activity, and dietary quality) [14,18,19], and three emerging factors (sleep duration, sedentary behavior, and social engagement) [20]. Social engagement and sedentary time were included because recent meta-analyses have demonstrated that they are independently associated with cognitive decline and dementia risk [21,22]. Smoking: Participants were classified as current or non-current smokers (former/never), with non-current status deemed favorable. Alcohol intake was quantified from self-reported consumption frequency and volume; moderate consumption (women ≤ 14 g/day; men ≤ 28 g/day) was considered favorable, with excess intake classified otherwise [23]. Physical activity was operationalized as moderate-to-vigorous activity (MVPA) minutes: moderate activity minutes plus twice vigorous activity minutes weekly; ≥150 MVPA minutes/week indicated favorable status [24]. Duration of moderate and vigorous physical activity was obtained from the UK Biobank baseline touchscreen questionnaire, which was based on the International Physical Activity Questionnaire (IPAQ) short form [25]. Dietary quality was evaluated based on cardiovascular health guidelines emphasizing whole grains, vegetables, fruits, seafood, dairy, and plant oils while limiting refined grains, sweetened beverages, and processed meats. Adherence to ≥ 6 components was classified as healthy [26]. Self-reported nightly sleep of 7–8 h was classified as favorable. Daily sedentary duration (computer use, television, driving) below 4 h indicated favorable status. Social engagement was gauged using three items: living alone, infrequent visits from relatives/friends (<once/month), and no weekly group activities. A score of 0–1 indicated healthy social contact; ≥2 indicated social isolation [27].We assessed multicollinearity among the seven lifestyle components using variance inflation factors (VIF). All VIF values were close to 1 (**Table S1**), indicating no significant multicollinearity. The correlations between components were also low (all *r* < 0.2), further supporting their inclusion as independent components.

To account for varying strengths of association between individual behaviors and AD, we constructed a weighted composite score [28]. Each lifestyle factor was dichotomized as healthy (1 point) or unhealthy (0 point), and its association with AD risk was evaluated using a separate Cox proportional hazards model adjusted for age, sex, ethnicity, educational attainment, TDI, family history of dementia, body fat percentage, APOE ε4 carrier status, and AD-PRS. The β coefficient from each model was multiplied by −1 (denoted as $\omega_{i}$) and used as the weight in the calculation of the weighted lifestyle score, ensuring that lifestyle factors showing a protective association with AD risk received positive weights, whereas factors showing a positive association with AD risk received negative weights. The weighted lifestyle score was then calculated as the weighted sum of all lifestyle factors:

$$S=\sum_{i}\omega_{i}L_{i}$$

where $L_{i}$ denotes the binary value (0 or 1) of the *i*th lifestyle factor. To standardize the score, it was rescaled to range from 0 to 7 as follows:

$$\text{Lifestyle}\;\text{score}=7\times \frac{S-S_{\text{min}}}{S_{\text{max}}-S_{\text{min}}}$$

where $S_{\text{min}}$ and $S_{\text{max}}$ represent the minimum and maximum values of the unstandardized weighted sum S.

This scoring method allows the integration of multiple lifestyle factors into a single, standardized measure, with the final score ranging from 0 to 7. Because the rescaling procedure is a linear min–max transformation, it preserves the relative magnitude and ordering of the weights across lifestyle factors and affects only the scale of the score rather than the proportional contribution of each component.

Higher scores indicate healthier lifestyle patterns. The final score was categorized into quintiles to define lifestyle categories: very unhealthy, unhealthy, intermediate, healthy, and very healthy. In sensitivity analyses, an unweighted lifestyle score was constructed by summing the seven factors (range 0–7) and categorized as 0–2, 3, 4, 5, and 6–7 points.

### 2.3 Assessment of hyperlipidemia and outcomes

Hyperlipidemia was ascertained by lipid-lowering medication use or baseline low-density lipoprotein cholesterol (LDL-C) ≥ 4.0 mmol/L, a threshold commonly used in population-based studies [29], and indicative of elevated cardiovascular risk in adult populations [30]. This threshold aligns with contemporary lipid management guidelines, where LDL-C is considered the primary therapeutic target [31].

AD was ascertained using UK Biobank Data-Field 42020 (date of Alzheimer’s disease report) [32], with cases defined by the presence of ICD-9 code 331.0 or ICD-10 codes F00, F00.0, F00.1, F00.2, F00.9, G30, G30.0, G30.1, G30.8, and G30.9. The algorithm employed in this study extracted any pre-defined ICD codes from hospital admission records (HES, APC, SMR01, and PEDW), regardless of whether they were recorded in primary or secondary diagnostic positions. Following a standardized methodology, the earliest chronological record across all available sources (self-reports, hospital admissions, and death registries) was identified and designated as the formal AD report date. The cutoff date for outcome ascertainment was October 30, 2023, coinciding with the timing of data extraction. Primary care records were not included in this algorithm. Since the algorithm itself does not inherently distinguish between pre-existing and new-onset conditions, incident cases were strictly restricted to those whose AD diagnosis post-dated their baseline time. Follow-up time was continued until the first instance of AD onset, mortality, or the study’s terminal date of October 30, 2023 [33].

### 2.4 Determination of covariates

Models incorporated age, sex, educational attainment, ethnicity, TDI, dementia family history, body mass index (BMI), APOE ε4 carrier status, and AD-PRS. Education was trichotomized (university degree = high; unlisted qualifications = low; remainder = medium). Ethnicity was dichotomized (White/non-White). TDI quantifies area-level socioeconomic disadvantage [34]. Family history of dementia required ≥ 1 affected first-degree relative. APOE ε4 carrier status was derived from rs7412/rs429358 genotypes. AD-PRS is a standardized score derived from AD-associated genetic loci, with higher scores indicating greater genetic susceptibility [35].

### 2.5 Statistical analysis

Descriptive statistics (means ± SD for continuous; counts with percentages for categorical variables) characterized the cohort overall and by hyperlipidemia status. Cox proportional hazards models estimated hazard ratios (HRs) and 95 % confidence intervals (CIs) relating lifestyle categories and individual behaviors to AD incidence. Sequential adjustment proceeded as: Model 1 (crude); Model 2 (demographics, socioeconomics, BMI); Model 3 (additionally family history, AD-PRS, APOE ε4). Model 3 served as primary model. The proportional hazards assumption was tested using Schoenfeld residuals for all Cox models and was satisfied for the main exposure (lifestyle categories). Associations between lifestyle categories and AD were evaluated separately in individuals with and without hyperlipidemia. Among those with hyperlipidemia, we further assessed associations for individual lifestyle factors. Age-stratified analyses (<50, 50–60, and >60 years) were conducted within the hyperlipidemia group. To evaluate potential effect modification, stratified analyses were performed by APOE ε4 carrier status and AD-PRS, with interaction terms tested using likelihood ratio tests (LRTs). Sensitivity analyses among individuals with hyperlipidemia included: (1) an unweighted lifestyle score assigning equal weights to all factors (range 0–7); (2) exclusion of individuals who developed AD within the first 2 years of follow-up; (3) additional adjustment for diabetes, depression, and hypertension, which are common clinical drivers of lipid-lowering therapy and help account for potential confounding by indication; (4) treatment of death as a competing risk using Fine–Gray models, with reporting of subdistribution hazard ratios (SHRs) and cumulative incidence functions (CIFs); and (5) exclusion of former drinkers.

## 3 Results

### 3.1 Characteristics of individuals

Of the 241,642 participants (mean age 56.3 ± 8.1 years; 49.2 % women), 104,082 (43.1 %) had hyperlipidemia at baseline (Table 1). The majority (96.1 %) were White. Compared with those without hyperlipidemia, individuals with hyperlipidemia were older, had greater BMI and TDI values, were less likely to have higher education, and had a greater proportion of males, family history of dementia, and APOE ε4 carriers. Mean AD-PRS was also higher in the hyperlipidemia group. During a median follow-up of 14.5 years (interquartile range: 13.8–15.2), 1728 participants developed AD, including 977 cases among those with hyperlipidemia.

**Table 1:** Baseline characteristics of the study population (*n* = 241,642).

Variable	Overall (*n* = 241,642)	Hyperlipidemia	
		No (*n* = 137,560)	Yes (*n* = 104,082)
Age, years	56.26 ± 8.08	54.57 ± 8.23	58.50 ± 7.30
Sex, n (%)			
Female	118,836 (49.20)	72,623 (52.8)	46,213 (44.4)
Male	122,806 (50.80)	64,937 (47.2)	57,869 (55.6)
BMI, kg/m^2^	26.94 ± 4.31	26.31 ± 4.28	27.78 ± 4.21
Education level, n (%)			
Low	73,879 (30.60)	41,519 (30.20)	32,360 (31.10)
Medium	66,498 (27.50)	36,160 (26.30)	30,338 (29.10)
High	101,265 (41.90)	59,881 (43.50)	41,384 (39.80)
TDI	−1.61 ± 2.87	−1.58 ± 2.88	−1.64 ± 2.86
ethnicity, n (%)			
White	232,250 (96.10)	131,779 (95.80)	100,471 (96.50)
Others	9392 (3.90)	5781 (4.20)	3611 (3.50)
APOE ɛ4 carrier status, n (%)			
No carrier	178,650 (73.90)	107,377 (78.10)	71,273 (68.50)
Carrier	62,992 (26.10)	30,183 (21.90)	32,809 (31.50)
Standard PRS for AD	0.05 ± 1.00	−0.02 ± 0.97	0.15 ± 1.03
Family History of dementia			
No	186,708 (77.30)	109,996 (80.00)	76,712 (73.70)
Yes	54,934 (22.70)	27,564 (20.00)	27,370 (26.30)

Data are mean ± SD or n (%). Abbreviations: BMI, Body mass index; TDI, Townsend deprivation index; APOE ε4, apolipoprotein E epsilon 4 allele; PRS, polygenic risk score; AD, Alzheimer’s disease.

### 3.2 Association of lifestyle factors and AD risk

Table 2 displays domain-specific associations. In fully adjusted models, non-current smoking (HR: 0.87; 95 % CI: 0.79–0.96; *P* = 0.006), regular MVPA (HR: 0.87; 95 % CI: 0.79–0.97; *P* = 0.009), sleep duration of 7–8 h/night (HR: 0.86; 95 % CI: 0.78–0.95; *P* = 0.003), and limited sedentary time (HR: 0.89; 95 % CI: 0.80–0.99; *P* = 0.037) each related to reduced AD risk. Contrary to expectations, moderate alcohol intake associated with 13 % higher risk versus excess consumption (HR: 1.13; 95 % CI: 1.02–1.24; *P* = 0.018). Diet and social contact showed no significant associations.

**Table 2:** Association between individual lifestyle factors and incidence of AD.

	Model 1		Model 2		Model 3	
	HR (95 % CI)	*P* Value	HR (95 % CI)	*P* Value	HR (95 % CI)	*P* Value
Smoking						
Current	Ref		Ref		Ref	
No current	0.69 (0.63, 0.76)	<0.001	0.87 (0.79, 0.96)	0.004	0.87 (0.79, 0.96)	0.006
Drinking						
Excessive	Ref		Ref		Ref	
Moderate	1.26 (1.14, 1.38)	<0.001	1.14 (1.04, 1.26)	0.008	1.13 (1.02, 1.24)	0.018
Diet						
Unhealthy	Ref		Ref		Ref	
Healthy	1.27 (1.15, 1.42)	<0.001	1.04 (0.94, 1.16)	0.439	1.02 (0.91, 1.13)	0.760
Physical activity						
Irregular	Ref		Ref		Ref	
Regular	0.99 (0.90, 1.10)	0.902	0.89 (0.80, 0.99)	0.024	0.87 (0.79, 0.97)	0.009
Sleep duration, h/day						
Non-optimal	Ref		Ref		Ref	
Optimal	0.80 (0.72, 0.88)	<0.001	0.86 (0.78, 0.95)	0.003	0.86 (0.78, 0.95)	0.003
Sedentary						
Yes	Ref		Ref		Ref	
No	0.74 (0.67, 0.82)	<0.001	0.88 (0.79, 0.98)	0.023	0.89 (0.80, 0.99)	0.037
Social contact						
Isolated	Ref		Ref		Ref	
Non-isolated	0.82 (0.64, 1.04)	0.096	0.93(0.73, 1.18)	0.528	0.91 (0.72, 1.16)	0.449

Model 1: unadjusted. Model 2: adjusted for age, sex, ethnicity, BMI, education, TDI, UKB center. Model 3: additionally adjusted for family history, APOE ɛ4 carrier status, AD-PRS, diabetes status, hypertension status, depression status. Abbreviations: AD, Alzheimer’s disease; BMI, Body mass index; TDI, Townsend deprivation index; UKB, UK Biobank; APOE ε4, apolipoprotein E epsilon 4 allele; PRS, polygenic risk score.

The weighted lifestyle score ranged from –1.02 to 5.98 (rescaled: 0–7). Based on quintiles, participants were categorized as very unhealthy (*n* = 5426), unhealthy (*n* = 29,531), intermediate (*n* = 69,719), healthy (*n* = 83,739), and very healthy (*n* = 53,227). Table 3 presents tier-specific associations. Relative to intermediate, unhealthy status related to elevated AD risk (HR: 1.17; 95 % CI: 1.02–1.35; *P* = 0.025), while healthy (HR: 0.86; 95 % CI: 0.76–0.96; *P* = 0.009) and very healthy (HR: 0.73; 95 % CI: 0.63–0.85; *P* < 0.001) tiers showed progressively lower risk. The very unhealthy tier lacked significant association, likely reflecting sparse events.

**Table 3:** Association between lifestyle categories and incidence of AD.

Lifestyle category	Model 1		Model 2		Model 3	
	HR (95 % CI)	*P* Value	HR (95 % CI)	*P* Value	HR (95 % CI)	*P* Value
Intermediate	Ref	Ref	Ref			
Very Unhealthy	1.08 (0.80, 1.46)	0.620	0.98 (0.73, 1.33)	0.910	0.98 (0.75, 1.41)	0.897
Unhealthy	1.27 (1.10, 1.45)	<0.001	1.18 (1.03, 1.36)	0.020	1.17 (1.01, 1.34)	0.025
Healthy	0.77 (0.69, 0.87)	<0.001	0.85 (0.75, 0.95)	0.005	0.85 (0.78, 0.99)	0.008
Very Healthy	0.59 (0.51, 0.68)	<0.001	0.74 (0.64, 0.86)	<0.001	0.74 (0.63, 0.85)	<0.001

Model 1: unadjusted. Model 2: adjusted for age, sex, ethnicity, BMI, education, TDI, UKB center. Model 3: additionally adjusted for family history, APOE ɛ4 carrier status, AD-PRS, diabetes status, hypertension status, depression status. Abbreviations: AD, Alzheimer’s disease; BMI, Body mass index; TDI, Townsend deprivation index; UKB, UK Biobank; APOE ε4, apolipoprotein E epsilon 4 allele; PRS, polygenic risk score.

**Fig. 2:**
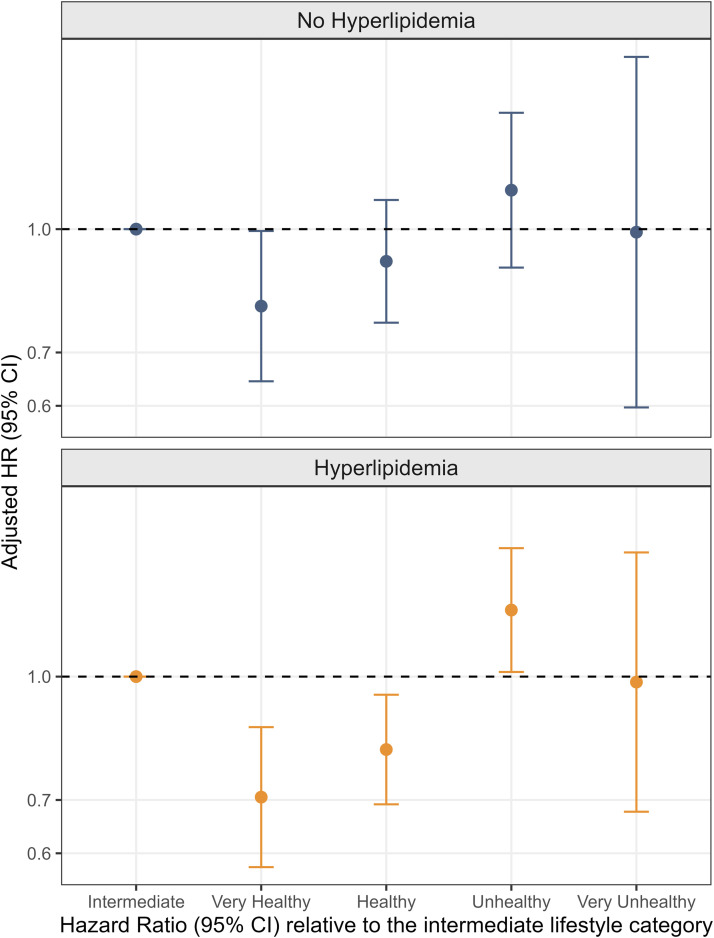
Association of lifestyle categories with AD risk in individuals with and without hyperlipidemia. Lifestyle categories group-specific AD cases in individuals with hyperlipidemia: Intermediate (*n* = 333), Very Healthy (*n* = 136), Healthy (*n* = 289), Unhealthy (*n* = 189), Very Unhealthy (*n* = 30). Lifestyle categories group-specific AD cases in individuals without hyperlipidemia: Intermediate (*n* = 242), Very Healthy (*n* = 129), Healthy (*n* = 251), Unhealthy (*n* = 113), Very Unhealthy (*n* = 16). Abbreviation: AD, Alzheimer’s disease.

Associations were then examined by hyperlipidemia status (Fig. 2). Among individuals with hyperlipidemia, compared with the intermediate category, healthy and very healthy lifestyles were associated with 19 % (HR: 0.81; 95 % CI: 0.69–0.95; *P* = 0.009) and 29 % (HR: 0.71; 95 % CI: 0.58–0.86; *P* < 0.001) lower AD risk, respectively. Among individuals without hyperlipidemia, a significant association was observed only in the very healthy lifestyle category (HR = 0.79; 95 % CI: 0.63–0.99; *P* = 0.042), whereas no significant associations were found for the other lifestyle categories. When lifestyle categories were additionally modeled as an ordinal variable (1 = very unhealthy, 5 = very healthy), a significant linear trend toward lower AD risk with healthier lifestyles was observed in both groups. The HR per lifestyle tier was lower in the hyperlipidemia group (HR = 0.85; 95 % CI: 0.80–0.91; *P* for trend < 0.001) than in the non-hyperlipidemia group (HR = 0.90; 95 % CI: 0.84–0.97; *P* for trend = 0.006).

### 3.3 Individual lifestyle factors and AD risk in hyperlipidemia

Within hyperlipidemic participants (**Figure S1**), never smokers showed significantly lower risk compared with current smokers (HR: 0.69; 95 % CI: 0.55–0.87; *P* = 0.002), while former smokers showed no significant association. Regarding sleep, ≥9 h nightly associated with 46 % elevated risk versus 7–8 h (HR: 1.46; 95 % CI: 1.20–1.76; *P* < 0.001); < 7 h showed no effect. Greater social engagement related to reduced AD risk.

Relative to the lowest social contact score, moderate social engagement showed no significant association (HR: 0.84; 95 % CI: 0.62–1.14; *P* = 0.262), whereas maximal social contact associated with significantly reduced risk (HR: 0.70; 95 % CI: 0.52–0.94; *P* = 0.017). Higher alcohol intake showed a trend toward reduced AD risk. Diet quality, physical activity, and sedentary time showed no significant associations.

### 3.4 Role of age at baseline in individuals with hyperlipidemia

We formally tested the interaction between age (continuous) and lifestyle categories using a Cox model, but the interaction terms were not statistically significant (*P* for LRT = 0.360). One likely reason is the distribution of AD events across age: younger participants (≤ 50 years old) had very few events, limiting the statistical power to detect interaction effects.

**Fig. 3:**
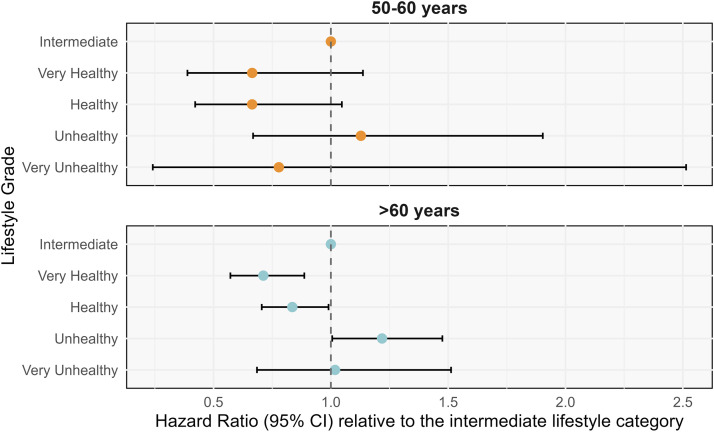
Association between lifestyle categories and AD risk across age groups in hyperlipidemia. Lifestyle categories group-specific AD cases in 50–60 years: Intermediate (*n* = 43), Very Healthy (*n* = 20), Healthy (*n* = 33), Unhealthy (*n* = 21), Very Unhealthy (*n* = 3). Lifestyle categories group-specific AD cases in >60 years: Intermediate (*n* = 288), Very Healthy (*n* = 115), Healthy (*n* = 255), Unhealthy (*n* = 166), Very Unhealthy (*n* = 27). Abbreviation: AD, Alzheimer’s disease.

Age-stratified analyses were conducted among participants with hyperlipidemia (Fig. 3). Among 14,819 individuals aged < 50 years, only six AD events occurred, yielding unstable estimates with extremely wide CIs; this age group was excluded from further analysis. Among 40,252 individuals aged 50–60 years (120 AD events), compared with the intermediate category, healthy (HR: 0.67; 95 % CI: 0.43–1.05; *P* = 0.081) and very healthy (HR: 0.67; 95 % CI: 0.39–1.15; *P* = 0.143) lifestyles showed approximately 33 % lower AD risk, though neither reached statistical significance.

Among 49,011 individuals aged >60 years (851 AD events), lifestyle categories were significantly associated with AD risk. Versus intermediate, healthy (HR: 0.83; 95 % CI: 0.70–0.99; *P* = 0.033) and very healthy (HR: 0.71; 95 % CI: 0.57–0.89; *P* = 0.003) tiers related to lower risk, while unhealthy status related to elevated risk (HR: 1.23; 95 % CI: 1.01–1.48; *P* = 0.006).

### 3.5 Joint effects of lifestyle and genetic predisposition in hyperlipidemia

Among individuals with hyperlipidemia, we examined whether genetic factors modified lifestyle-AD associations (**Figure S2**). Lifestyle-by-APOE ε4 interaction terms were tested by including cross-product terms in fully adjusted Cox proportional hazards models. A statistically significant interaction between lifestyle and APOE ε4 status was observed (*P* for LRT = 0.020). In contrast, no significant interaction was detected between lifestyle and AD polygenic risk score (*P* for likelihood ratio test = 0.210). Genetic interaction test results were presented in **Table S2**.

### 3.6 Sensitivity analysis

**Fig. 4:**
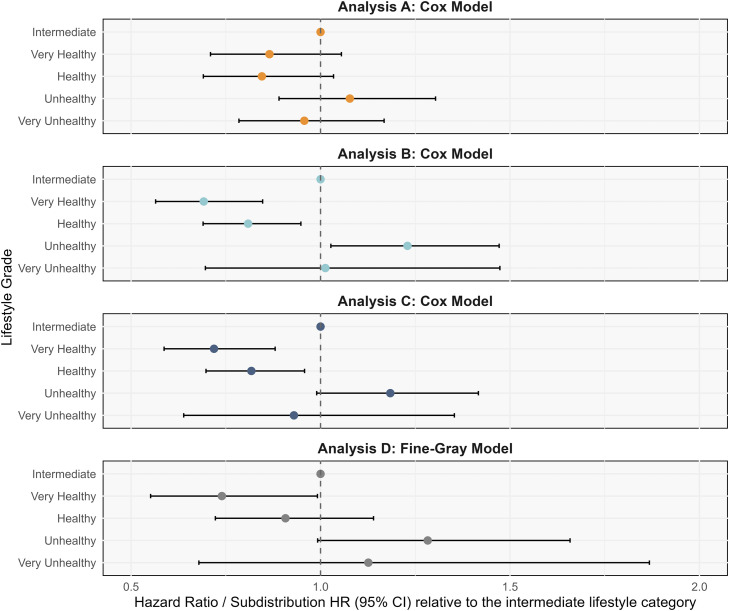
Sensitivity analyses. Analysis A: Analysis based on re-scoring seven lifestyle factors with equal weights. Analysis B: Analysis excluding participants who developed dementia within two years of follow-up. Analysis C: Analysis additionally adjusting for diabetes, hypertension, and depression as covariates. Analysis D: Analysis considering death as a component risk.

Sensitivity analyses corroborated primary findings (Fig. 4). Associations were consistent after excluding early-onset dementia cases (within two years), with additional comorbidity adjustment (diabetes, hypertension, depression). In sensitivity analyses using an unweighted lifestyle score, the associations with AD risk were directionally consistent with those observed for the weighted score, with similar trends across lifestyle categories. Although some estimates were attenuated and did not reach statistical significance, the overall pattern of results and the main conclusions were unchanged. In the competing risk analysis, Fine–Gray models indicated a significantly lower subdistribution hazard of AD in the very healthy lifestyle category (SHR = 0.77, 95 % CI: 0.66–0.89), which was consistent with the separation of CIF curves (**Figure S3**). After excluding former drinkers, the association between alcohol consumption and AD risk (**Figure S4**) remained essentially unchanged. The association between lifestyle categories and AD risk (**Table S3**) was consistent with the main analysis.

## 4 Discussion

This large UK Biobank cohort study of over 240,000 participants demonstrated that healthier lifestyle patterns substantially reduced AD risk, particularly among individuals with hyperlipidemia. AD risk increased with unhealthy behaviors and decreased progressively with healthier patterns. Hyperlipidemic individuals maintaining very healthy lifestyles were associated with approximately 30 % lower AD risk, an association most pronounced among those exceeding 60 years. These associations were consistent across strata of genetic susceptibility and remained robust in sensitivity analyses.

Our findings align with previous work demonstrating that composite healthy lifestyle indices are associated with reduced dementia risk [14,15,[36], [37], [38]]. However, few studies have focused on individuals with hyperlipidemia despite their elevated risk [4,38]. By stratifying by hyperlipidemia status, we provide novel evidence that healthy lifestyle may be particularly important in this high-risk group. Protective associations were primarily observed among hyperlipidemic individuals, whereas associations were weaker and less consistent among those without hyperlipidemia., raising the possibility that hyperlipidemia marks a state of heightened vulnerability to lifestyle factors. Age-stratified analyses further suggest that healthy lifestyle remains beneficial even at older ages, consistent with evidence that mid-to-late life lifestyle modification can delay dementia onset [[38], [39], [40]].

Domain-specific findings largely accorded with established literature. Tobacco abstinence, regular exercise, and adequate sleep were each associated with lower AD risk [13,41], while long sleep (≥9 h) was associated with higher risk among hyperlipidemic individuals [36,42]. Social contact showed a threshold effect, with only the highest level of engagement appearing protective [27]. The association between moderate alcohol intake and higher AD risk compared to excessive drinking was unexpected. This finding may reflect several factors: First, the “sick quitter” phenomenon, where individuals with health problems (including early cognitive decline) may reduce or stop alcohol consumption, potentially biasing the moderate drinking group. Second, residual confounding by unmeasured factors associated with both drinking patterns and AD risk cannot be excluded [43]. Previous studies have reported similar paradoxical findings [44,45]. We caution against causal interpretation of this association and highlight the need for further investigation using more detailed lifetime drinking histories and longitudinal assessments. We did not observe significant associations for diet or sedentary behavior, possibly reflecting limited sensitivity of baseline metrics or regression dilution over time [26].

APOE ε4, the predominant AD genetic determinant, may interact with hyperlipidemia [46]. Lifestyle benefits appeared attenuated among hyperlipidemic APOE ε4 carriers, suggesting intensified interventions may be required. Mechanistically, hyperlipidemia disrupts Aβ production and clearance, promotes abnormal tau phosphorylation, and contributes to neuroinflammation and oxidative stress [[47], [48], [49]]. Healthy lifestyle interventions that manage hyperlipidemia therefore represent a feasible strategy to reduce both cardiovascular and neurodegenerative risks [50].

Weaker associations between lifestyle categories and AD risk were observed among individuals without hyperlipidemia, with a significant association observed only for the very healthy lifestyle category. Several explanations may account for this pattern. First, the incidence of AD was lower in the non-hyperlipidemia group (0.55 %) than in the hyperlipidemia group (0.94 %). Second, hyperlipidemia may reflect a state of underlying metabolic vulnerability, and broader metabolic dysregulation has been implicated in AD pathogenesis in both epidemiological and mechanistic studies [51]. In this context, unhealthy lifestyle factors may exacerbate lipid abnormalities and related metabolic disturbances, whereas healthier lifestyles may mitigate these processes, leading to more pronounced risk reductions. By contrast, among individuals without hyperlipidemia, lower baseline metabolic risk may attenuate the observable impact of lifestyle factors on AD risk.

Several limitations warrant consideration. Because hyperlipidemia was partly ascertained based on lipid-lowering medication use, particularly statins, the potential influence of statin therapy on the observed associations cannot be entirely excluded. Observational design precludes causal inference, and unmeasured confounding cannot be excluded. Single baseline assessments of hyperlipidemia and lifestyle may introduce misclassification biasing toward null. Lifestyle behaviors and lipid measures were assessed only at baseline, and changes over time were not captured. Such within-person variability may lead to regression-dilution bias, which would be expected to attenuate associations toward the null. Consequently, the observed associations may underestimate the true long-term relationships between lifestyle factors, lipid status, and AD risk. Future studies with repeated measurements are needed to address this limitation. AD diagnoses from electronic health records may include misclassification, and competing risk of death was not modeled. The UK Biobank comprises predominantly White, healthier volunteers, limiting generalizability [16,17]. Participants with better cognitive function and healthier lifestyles may be more likely to enroll in long-term studies, potentially introducing selection bias. Furthermore, the observed associations may not be directly applicable to populations from different ethnic backgrounds, who may have distinct genetic susceptibility, lifestyle patterns, and AD risk profiles. Replication in more ethnically and clinically diverse cohorts is warranted. Finally, multiple subgroup analyses were conducted without formal multiplicity adjustment; subgroup results should be interpreted as exploratory.

Within this large UK Biobank prospective cohort, adherence to favorable lifestyle practices related to diminished AD risk, with particularly pronounced benefits among individuals with hyperlipidemia and those aged > 60 years. Among hyperlipidemic adults, AD risk decreased progressively with increasing healthy lifestyle factors and was substantially reduced among those adhering to most or all the seven favorable behaviors. These findings support comprehensive lifestyle modification as a strategy to mitigate AD risk for hyperlipidemic populations and underscore the value of integrating brain-health considerations into cardiovascular prevention programs.

## Abbreviations

AD: Alzheimer's disease

Aβ: amyloid-β plaques

APP: amyloid precursor protein

APOE: Apolipoprotein E

GWAS: Genome-wide association study

AD-PRS: AD polygenic risk score

TDI: Townsend deprivation index

MVPA: moderate-to-vigorous activity

IPAQ: the International Physical Activity Questionnaire

VIF: variance inflation factors

LDL-C: Low-Density Lipoprotein Cholesterol

BMI: body mass index

HR: hazard ratio

CI: confidence interval

SHR: subdistribution hazard ratio

CIF: cumulative incidence function

LRT: likelihood ratio test

## Funding source

This study was supported by Health and Medical Scientific Research Project of Shenzhen Bao'an Medical Association (BAYXH2024001 to Yuzhong Xu).

## Ethics declaration

The UK Biobank was approved by the North West Multi-centre Ethics Committee, and written informed consent was provided by all participants. This study was conducted under UK Biobank project number 106528 in accordance with the Declaration of Helsinki. Written informed consent was obtained from all participants.

## Data availability

The usage of UK Biobank data has been approved by UK Biobank Research Team (Application ID: 106,528). Data from UK Biobank (https://www.ukbiobank.ac.uk/) are available on application.

## Declaration of the use of generative AI and AI-assisted technologies

During the preparation of this manuscript, the authors used ChatGPT (OpenAI, version 5.2) to assist with English language editing and improvement of clarity and grammar. The AI tool was not used to generate scientific content or interpret results. All scientific content and conclusions were developed and verified by the authors, who take full responsibility for the manuscript.

## CRediT authorship contribution statement

**Danyang Sun:** Writing – original draft, Methodology, Formal analysis. **Linling Yu:** Writing – review & editing, Supervision, Conceptualization. **Chenqi Liao:** Methodology. **Yuzhong Xu:** Writing – review & editing, Supervision, Conceptualization. **Wei Liu:** Supervision, Conceptualization. **Xiong Wang:** Writing – review & editing, Supervision, Project administration, Funding acquisition, Conceptualization.

## Declaration of competing interest

The authors declare that they have no known competing financial interests or personal relationships that could have appeared to influence the work reported in this paper.

## Appendix Supplementary materials

Image, application 1
