# Supplementary material for: Healthy lifestyle and Alzheimer’s disease in individuals with hyperlipidemia: A prospective cohort study
Source: J Prev Alzheimers Dis. 2026 Feb 27;13(4):100520. doi: 10.1016/j.tjpad.2026.100520 (PMC12964015; doi:10.1016/j.tjpad.2026.100520)
Supplement: Supplementary file 1 [file mmc1.docx]

**Healthy lifestyle and Alzheimer’s disease in individuals with hyperlipidemia: a prospective cohort study**

**Table S1. VIF for the seven lifestyle factors.**

| Lifestyle factor | VIF |
| --- | --- |
| Smoking | 1.03 |
| Drinking | 1.03 |
| Diet | 1.01 |
| Physical activity | 1.01 |
| Sleep duration | 1.00 |
| Sedentary | 1.01 |
| Social contact | 1.00 |

Abbreviation: VIF, Variance Inflation Factor.

**Table S2. Genetic interaction test results.**

| Lifestyle categories × Interaction term | Overall interaction test | Chi² | df | *P* for interaction |
| --- | --- | --- | --- | --- |
| Lifestyle categories × APOE ε4 | LRT | 12.54 | 4 | 0.021 |
| Lifestyle categories ×AD-PRS |  | 9.567 | 8 | 0.210 |

Abbreviations: AD, Alzheimer’s disease; APOE ε4, apolipoprotein E epsilon 4 allele; AD-PRS, polygenic risk score for Alzheimer’s disease; LRT, likelihood ratio test; Chi², Chi-square.

**Table S3. Association between lifestyle categories and incidence of AD excluding former drinkers.**

| Lifestyle category | Model 1 | | Model 2 | | Model 3 | |
| --- | --- | --- | --- | --- | --- | --- |
|  | HR (95 % CI) | *P* Value | HR (95 % CI) | *P* Value | HR (95 % CI) | *P* Value |
| Intermediate | Ref | | Ref | | Ref | |
| Very Unhealthy | 1.05 (0.73, 1.53) | 0.786 | 0.99 (0.68, 1.45) | 0.975 | 0.98 (0.68, 1.43) | 0.934 |
| Unhealthy | 1.27 (1.06, 1.52) | 0.009 | 1.22 (1.02, 1.45) | 0.033 | 1.21 (1.01, 1.45) | 0.035 |
| Healthy | 0.77 (0.66, 0.90) | 0.001 | 0.81 (0.69, 0.95) | 0.009 | 0.81 (0.69, 0.95) | 0.009 |
| Very Healthy | 0.65 (0.53, 0.80) | <0.001 | 0.72 (0.59, 0.88) | 0.002 | 0.71 (0.58, 0.86) | <0.001 |

Model 1: unadjusted. Model 2: adjusted for age, sex, ethnicity, BMI, education, TDI, UKB center. Model 3: additionally adjusted for family history, APOE ɛ4 carrier status, AD-PRS, diabetes status, hypertension status, depression status. Abbreviations: AD, Alzheimer’s disease; BMI, Body mass index; TDI, Townsend deprivation index; UKB, UK Biobank; APOE ε4, apolipoprotein E epsilon 4 allele; PRS, polygenic risk score.


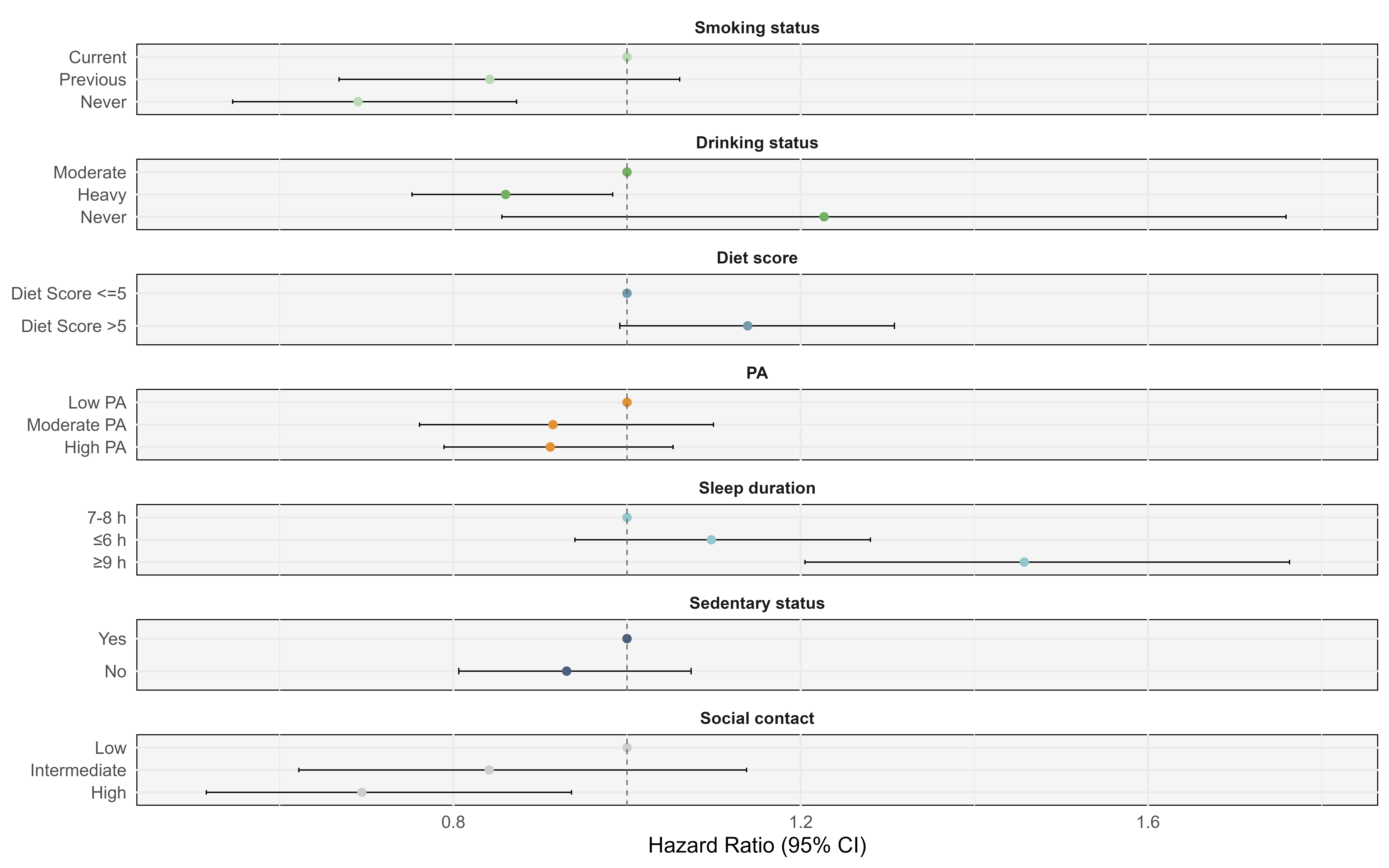


**Figure S1. Association of individual lifestyle factors with AD risk in individuals with hyperlipidemia.** Abbreviations: AD, Alzheimer’s disease; PA, physical activity.


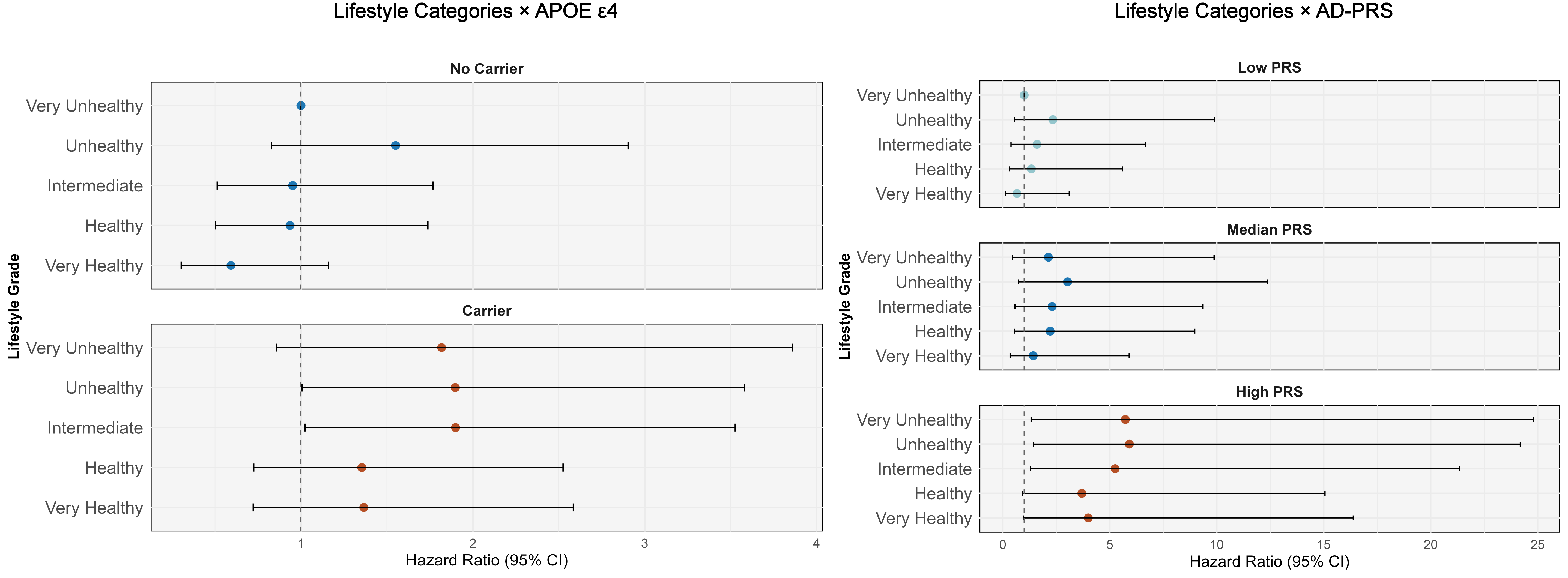


**Figure S2. The joint effects of lifestyle and APOE ɛ4 carrier status and PRS on the risk of AD incidence in Hyperlipidemia.** Abbreviations: AD, Alzheimer’s disease; APOE ε4, apolipoprotein E epsilon 4 allele; PRS, polygenic risk score.


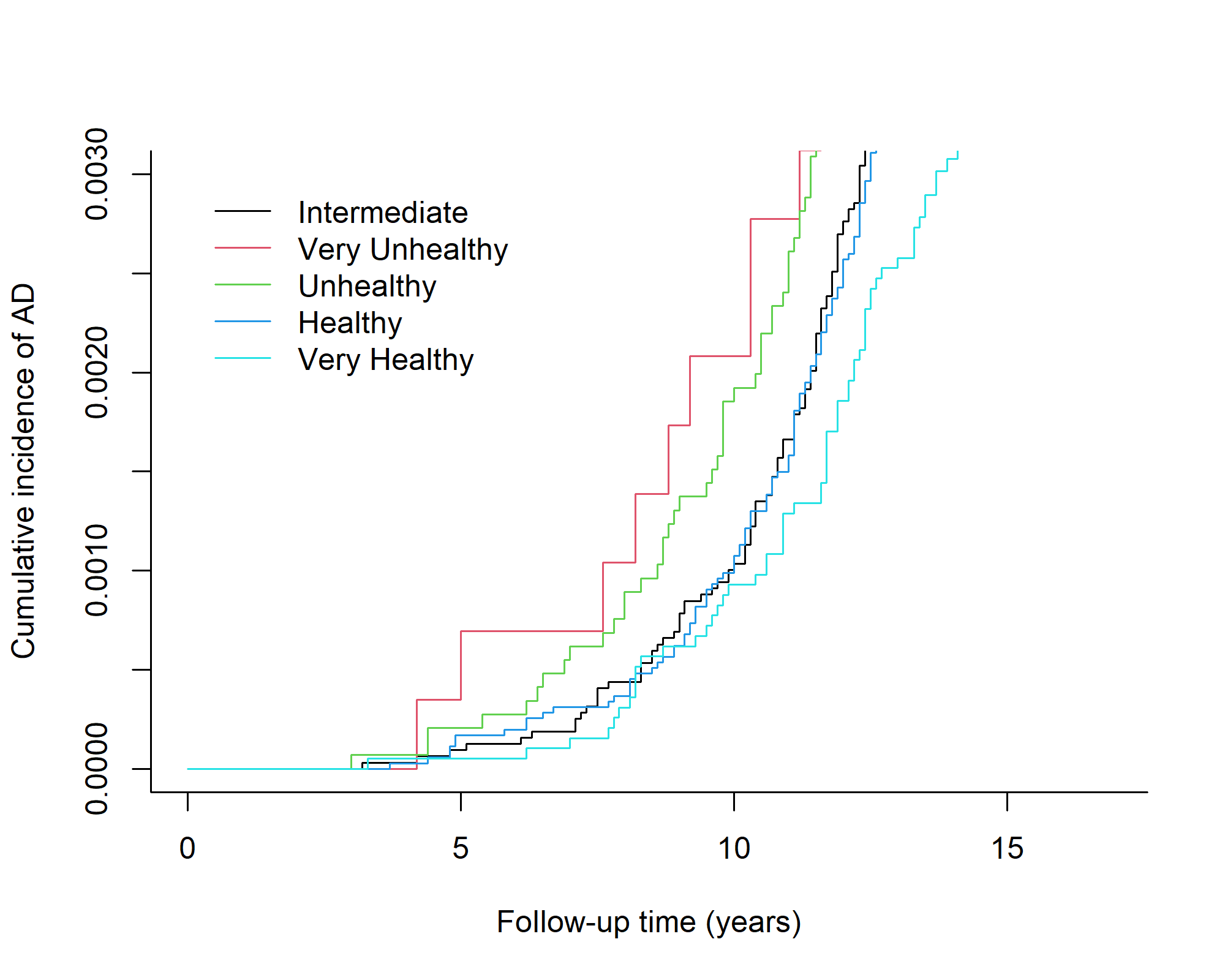


**Figure S3. CIF curves for AD across lifestyle categories among individuals with hyperlipidemia, accounting for the competing risk of death.** Abbreviations: CIF, cumulative incidence function; AD, Alzheimer’s disease.

**
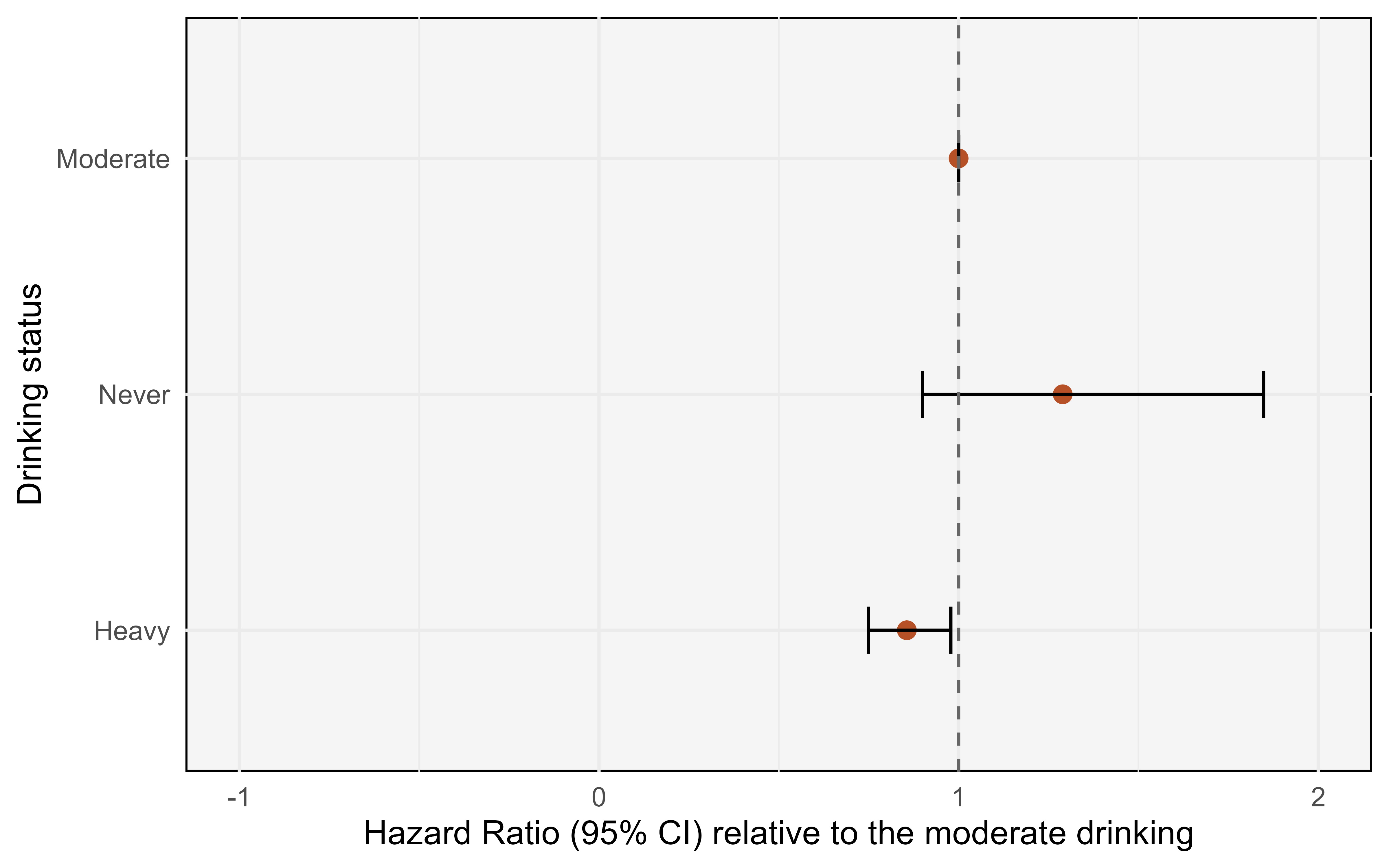
**

**Figure S4. Association between alcohol consumption and AD risk excluding former drinkers in hyperlipidemia.** Abbreviation: AD, Alzheimer’s disease.
